# Controlling the flux of reactive species: a case study on thin film deposition in an aniline/argon plasma

**DOI:** 10.1038/s41598-020-72634-y

**Published:** 2020-09-28

**Authors:** D. Sciacqua, C. Pattyn, A. Jagodar, E. von Wahl, T. Lecas, T. Strunskus, E. Kovacevic, J. Berndt

**Affiliations:** 1 GREMI UMR 7344, CNRS/Université D’Orléans, 45067 Orléans, France; 2 Chair for Multicomponent Materials, Institute for Materials Science, Kiel University, Kiel, Germany

**Keywords:** Applied physics, Plasma physics

## Abstract

The plasma based synthesis of thin films is frequently used to deposit ultra-thin and pinhole-free films on a wide class of different substrates. However, the synthesis of thin films by means of low temperature plasmas is rather complex due to the great number of different species (neutrals, radicals, ions) that are potentially involved in the deposition process. This contribution deals with polymerization processes in a capacitively coupled discharge operated in a mixture of argon and aniline where the latter is a monomer, which is used for the production of plasma-polymerized polyaniline, a material belonging to the class of conductive polymers. This work will present a particular experimental approach that allows to (partially) distinguish the contribution of different species to the film growth and thus to control to a certain extent the properties of the resulting material. The control of the species flux emerging from the plasma and contributing to the film growth also sheds new light on the deposition process, in particular with respect to the role of the ion component. The analysis of the produced films has been performed by means of Fourier Transform Infrared spectroscopy (FTIR) and Near Edge X-ray Absorption Fine Structure spectroscopy (NEXAFS).

## Introduction

Low temperature plasmas with their unique non-equilibrium properties are frequently used in industrial processes for the deposition of thin films or the activation of surfaces^1−4^. They offer fast, environment friendly and scalable possibilities for the mass fabrication of new materials and products. Moreover, plasma polymerization is a technique in particular suitable for the production of extremely uniform, ultrathin and pinhole-free layers that strongly adhere to a great class of different substrates^5^. A further advantage of this technique is that it eliminates various steps needed in more conventional coating processes. In addition it is solvent-free and does not involve, in contrast to many other methods, the use of costly chemicals.

An interesting example of the application of Plasma Enhanced Chemical Vapor Deposition (PECVD) is the plasma deposition of conductive polymers. Conductive polymers have attracted a great interest in recent years due to their unique properties, which make them suitable for a wide range of applications in electronics, optoelectronics and photonics^6,7^. Among the conductive polymers, polyaniline emerges: it has a good environmental stability and is easy to obtain starting from an inexpensive monomer. Moreover, it can be efficiently doped with different kinds of anions (I, Cl) or organic acids in order to improve its conductivity^8−11^. The main applications concern the production of light emitting diodes^12−14^, photovoltaics^15,16^, electromagnetic shielding^17^, and anticorrosion coatings for metals^18^, sensors^19,20^, super capacitors and energy storage devices^21−24^. The physical, chemical and morphological properties of conductive polymers in general and polyaniline in particular strongly depend on their composition and on the synthesis method^25^. To date, several methods are known in literature as e.g. chemical polymerization^26^, electrochemical polymerization^27^ and last but not least plasma polymerization^8,25,28−31^.

In plasma polymerization processes the electron impact dissociation of the original monomer is responsible for the initial formation of various species including neutral radicals as well as positive and negative ions. These species can induce subsequent polymerization reactions leading to the formation of larger oligomers^28,32−35^ and, depending on the process parameters, eventually even to the formation of nanoparticles^28,29,36−38^. All these species, which are formed in the plasma volume, contribute to the flux of species impinging on the substrate effecting the film growth in a synergistic way (see e.g.^39,40^ for the role of positive ions in the deposition of thin films from various precursors^41,42^, for the discussion of synergistic effects and their importance for radical induced film growth^43^, for the potential role of negative ions in pulsed plasmas and^44^ for the possible contribution of nanoclusters to the film growth in silane plasmas). The exact composition of the species flux for a given precursor, and thus the properties of plasma produced thin polymer films, generally depends on a great number of possible external parameters as e.g.: process pressure, electrode distance, gas mixture ratios (noble gas component/monomer), power, pulse frequency, duty cycle or ratio input flow rate/pumping speed (residence time)^45^. The aim of this contribution concerns the study of thin film deposition in an aniline/argon plasma and the possible control of the species flux, and thus the film properties by using a specific structured electrode later referred to as “channel electrode”. Although the results of this work refer to the aniline chemistry, the conception of the experiment can easily be transferred not only to other scientific setups but also to industrial applications. The characterization of the deposited films is done by profilometry, ellipsometry, FTIR and NEXAFS spectroscopy.

## Experimental set up

**Figure 1:**
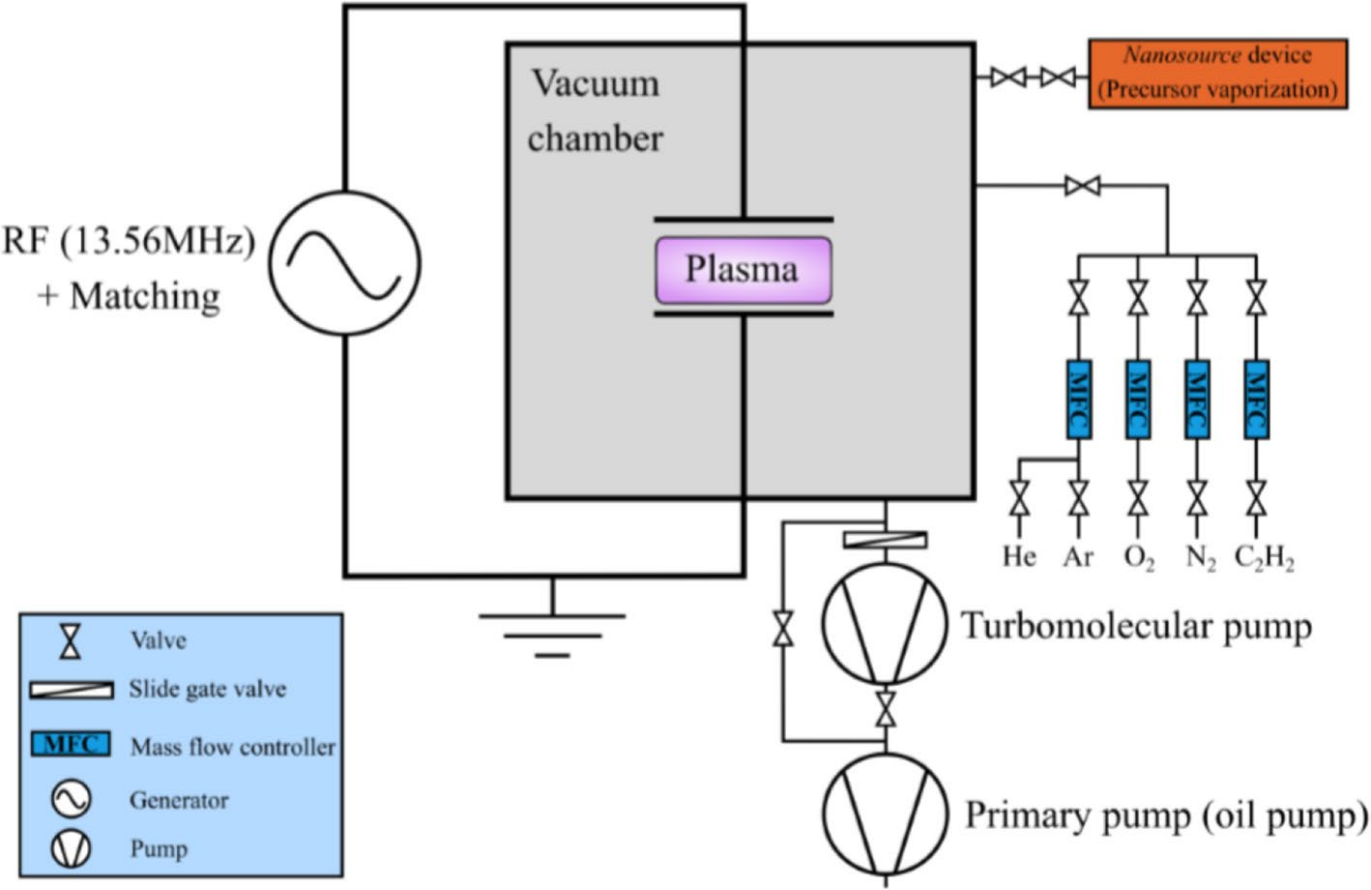
Scheme of the experimental set up.

The thin films are deposited in a capacitively coupled discharge (13.56 MHz) operated in an aniline/argon mixture. The experimental set up is shown in Fig. 1 and consists of a custom-build cylindrical reactor made of a large stainless steel chamber of 70 cm diameter and 72 cm height coupled with a vacuum pumping system (primary and turbomolecular).

The RF-electrodes consist of stainless steel with a diameter of 12 cm and are separated by 5.5 cm, where the powered electrode is the top one. The monomer vapor is generated from a liquid aniline precursor (Sigma Aldrich 242284) and is introduced into the reaction chamber through an evaporator, the NanoSource device developed by Omicron Technologies. The plasma-polymerized polyaniline deposition starts with pumping down the vacuum chamber to 10^−6^ mbar before each process and subsequently with introducing 20 sccm of argon (carrier gas) and 1 sccm of aniline. The working pressure is stabilized at 0.18 mbar in all experiments. The input average power is set to about 10 W and the discharge is pulsed at 25 Hz with a duty cycle of 0.5. These parameter (gas mixing ratio and pulsing frequency) are chosen in order to suppress the formation of nanoparticles within the plasma volume and to^28^ and to avoid in this way the contamination of our substrate with nanoparticles.

**Figure 2:**
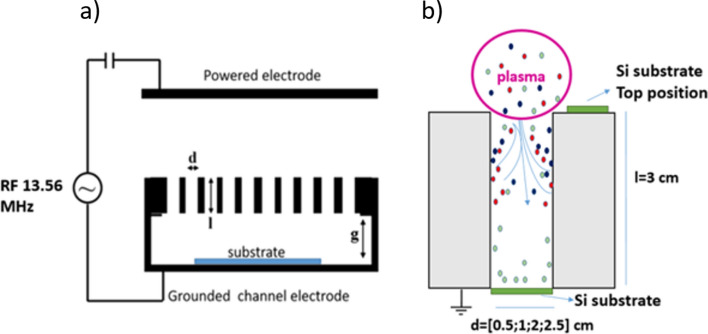
(**a**) General scheme of the channel electrode and (**b**) magnification of a single channel. The substrates are positioned directly below the holes (distance between sample and electrode g = 0 cm). In the experiment presented here, the metal plate with a thickness of l = 3 cm has only 4 holes/channels with diameters of 0.5, 1, 2 and 2.5 cm. To evaluate the effect of the holes, one reference wafer is placed on top of the metal plate during the experiments.

Figure 2 shows a sketch of the “channel electrode” which is used to control—to a certain extent—the flux of species reaching the substrate. The electrode consists of a metal plate, characterized by its thickness *l* and by holes/channels, which are drilled into the metal plate, characterized by their diameter *d*.

It is shown in^46^ that structured electrodes can have a tremendous influence on the electron heating dynamics and charge carrier density profiles in capacitively coupled radio frequency plasmas (above and inside the electrode structures). The investigations carried out in^46^ were performed in neon. In the present case the situation is more complex due to the reactive nature of the used gas mixture. In this case the sticking of reactive species to the side walls of the hole can add an additional filtering effect: species with a high sticking coefficient have smaller probabilities to reach the bottom of the hole and thus cannot contribute to the growth of the film at the bottom. The filter effect can be controlled by the aspect ratio of the hole, plate thickness *l* to diameter *d*. To guarantee a homogeneous film deposition the substrate can be separated from the holes by a certain distance *g*. In the present experiment, the metal plate has a thickness of *l* = 3 cm and features only 4 holes but with different diameters (*d* = 0.5 cm, 1.0 cm, 2.0 cm and 2.5 cm). The silicon wafers are attached to the metal plate directly beneath these holes (*g* = 0 cm).

The material characterization of the resulting thin films is realized by NEXAFS spectroscopy performed at the synchrotron facility of BESSY II (Helmholtz Zentrum Berlin, Germany) at the HESGM beamline. The spectra are taken in total electron yield using a self-built double channel-plate electron detector. The FTIR analysis of the deposited films is performed ex-situ, using a Bruker Vertex 70 Fourier-Transform Infrared Spectrometer. The film thickness is measured by ex-situ ellipsometry (Horiba UVisel 1, using a Cauchy dispersion model) and by means of a profilometer (Bruker DektaK). The plasma itself is monitored during the experiments by means of microwave interferometry, a method that delivers information about the electron density. These measurements of the electron density are performed using a S-band microwave interferometer MWI 2650-L (see also^47^).

## Experimental results and discussion

**Figure 3:**
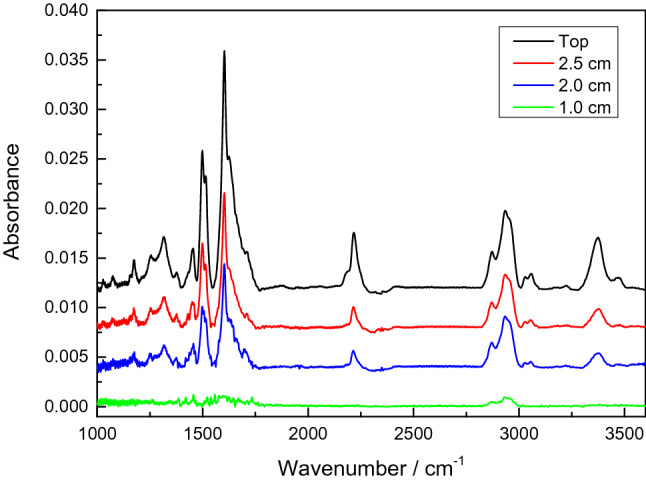
Ex-situ FTIR spectra of samples placed in different positions during the growth process. The black line shows the spectrum of the sample placed on top of the electrode. The red, blue and green line show spectra of samples placed under the holes with 2.5 cm, 2 cm and 1 cm diameter, respectively.

Figure 3 shows the ex-situ FTIR spectra of thin films from samples placed beneath the different holes. The spectrum in black refers to the sample placed on top of the electrode (see Fig. 2b).

The band around 3362 cm^−1^ can be associated to N–H vibrations^8,25,31,36,48^. The next two bands around 3020 cm^−1^ and 2930 cm^−1^ can be referred to C–H stretching modes in aromatic structures^48−50^ or to C–H stretching modes related to aliphatic compounds^8,31,49,50^. The peak around 2217 cm^−1^ can be attributed to C≡N bonds^49−51^. At 1496 and 1600 cm^−1^ the typical absorption peaks for C=C stretching of benzenoid and quinoid rings are observed^8,25,49,52,53^. The bands at 1173 cm^−1^ and at 1316 cm^−1^ arise from C–C and C=C interactions and C–N stretching^31,53^. The band at 1452 cm^−1^ can be attributed to C–H deformation^54^.

The spectra in red, blue and green in Fig. 3 represent the spectra of films from samples that are placed under the different holes of the structured electrode. Compared to the sample placed on top of the electrode, these spectra exhibit an overall decrease in absorbance. This reduction is the stronger, the smaller the size of the hole under which the sample is placed. It can be explained by a decrease in film thickness of the corresponding samples. The growth rate, measured by means of ellipsometry and profilometry, reduces from 4.0 nm/min for the sample placed on top to 0.25 nm/min for the sample placed under the 1 cm hole. Besides the overall decrease of the absorbance, the relative intensities of the individual bands with respect to each other are also changing. The peaks referred to benzenoid (1496 cm^−1^), quinoid (1600 cm^−1^), C≡N (2217 cm^−1^) and C–H (2930 cm^−1^) show a rather similar relative decrease – when going from the top to the bottom in Fig. 2. In comparison the decrease in absorbance at 2930 cm^−1^ (C–H stretching) is much less pronounced. These results show that, besides the growth rate, also the chemical structure of the thin films is altered depending on the size of the hole.

**Figure 4:**
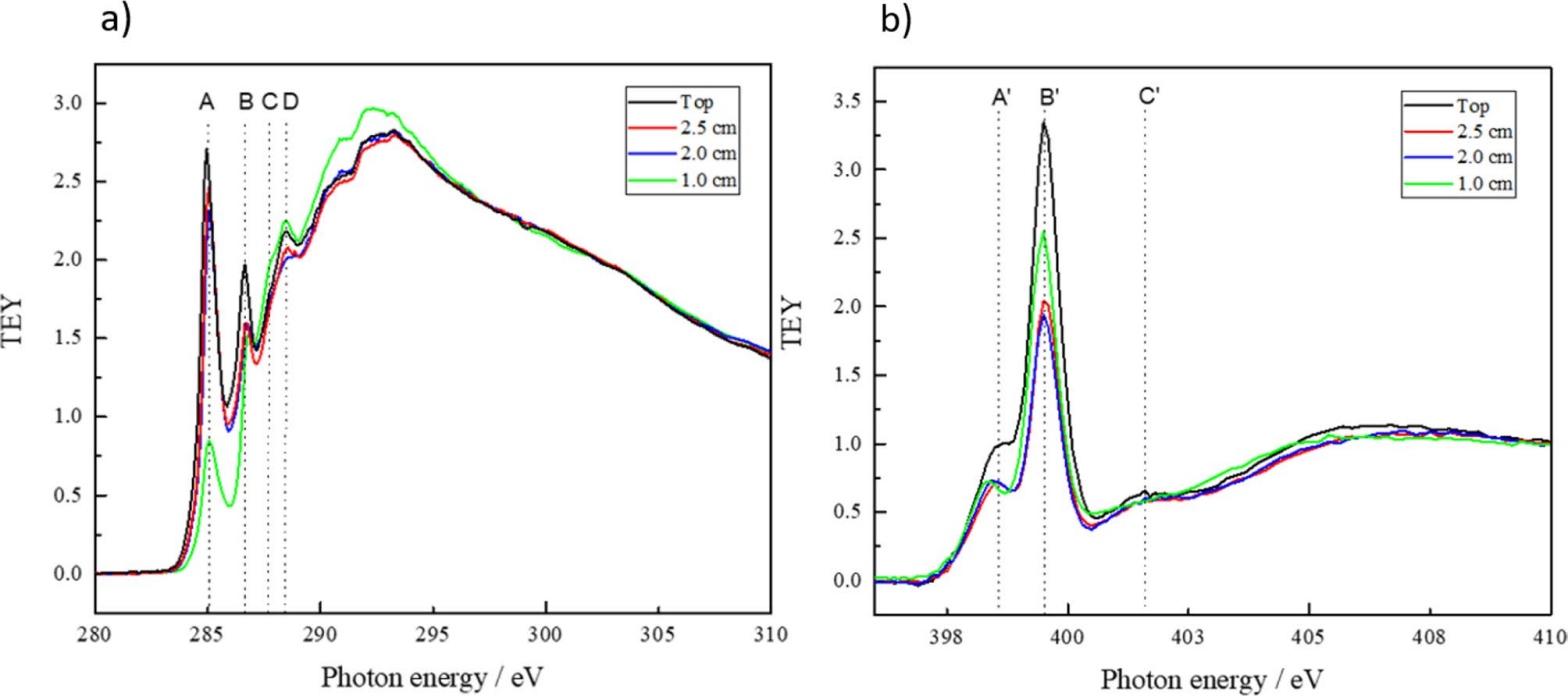
(**a**) NEXAFS C K-edge and (**b**) N K-edge spectra of thin films deposited on silicon samples placed in different positions: the black line shows the spectrum of the sample placed on top of the electrode, the red, blue, and green line show the spectra of the samples placed below the holes with diameters of 2.5 cm, 2.0 cm, and 1.0 cm, respectively.

This is confirmed by the NEXAFS spectra of the C K-edge and N K-edge shown in Fig. 4a,b. The spectra are acquired in total electron yield (TEY) mode. After acquisition the spectra are normalized to the photon flux by division through the transmission spectrum obtained on a freshly sputtered Au sample. NEXAFS is particular suitable to study systems with conjugated π-electron systems. In NEXAFS, changes in unsaturation are directly reflected in changes in intensities of the respective π*-resonances observed at the K-edge for each element.

The C K-edge spectrum of the sample placed on top (black) shows a sharp resonant feature at 285 eV (A) which is attributed to the C 1s → π* resonance of C=C bonds in aromatic systems^43,55−59^. This resonance is typical for sp^2^ hybridized carbon atoms in e.g. aromatic compounds and allows to assess the degree of unsaturation in carbonaceous compounds. The second resonance peak localized at around 286.8 eV (B) can be attributed to the C 1s → π* transition of carbon atoms bound to nitrogen^43,55−59^. Around 287.7 eV (C) is a small resonance indicating the presence of Rydberg states of C–H. The fourth resonance around 288.5 eV (D) can be related to the C 1s → π* resonance transition of C=O^60,61^. The broad bands above 290 eV are due to the C 1s → σ* transitions of different bonding environments (C–C, C–N, C–O)^60,61^.

Figure 4 shows that a reduction of the hole size leads to a strong decrease of the resonance peak at 285 eV. This indicates a loss of the aromatic character and is in agreement with the FTIR spectra in Fig. 3. The by far lowest intensity of this resonance appears for the sample placed under the 1 cm hole, where also the FTIR spectrum shows the almost complete disappearance of the aromatic structure. Correspondingly, the σ* resonances around 294 eV displays the highest intensity for the sample placed under the smallest hole. As expected, for these resonances the intensity is increasing with decreasing hole size. The Rydberg states, localized around 287.7 eV and indicating the contribution of C–H in the material, can be related to the FTIR peak around 2930 cm^−1^ corresponding to aliphatic C–H. In agreement with the FTIR results the resonance peak at 287.7 eV is the strongest for the sample placed below the 1 cm hole.

No clear trend can be stated on the other hand for the resonance around 286.8 eV due to multiple contributions of many carbon–nitrogen species in the material, e.g. imine, nitrile and amine which have to be further analyzed in the N K-edge. Here, Fig. 4b, the resonance near 398.6 eV (A′) corresponds most likely to the N 1 s → π* resonance of imine-like N-species^55,62,63^ while the intense resonant feature around 399.6 eV (B′) can be attributed to the N 1s → π* transition of nitrile groups (–C≡N). The less intense peak around 401.8 eV (C′) is due to the N 1s → π* transition of amine. While the small amine resonance peak at 401.8 eV is observed for the samples placed on top and in the 2.5 and 2.0 cm holes, the sample placed in the smallest hole of 1 cm does not show any significant presence of this feature.

However, the identification of the peaks is non-trivial due to several possible resonances originating from nitrile groups^63^.

**Figure 5:**
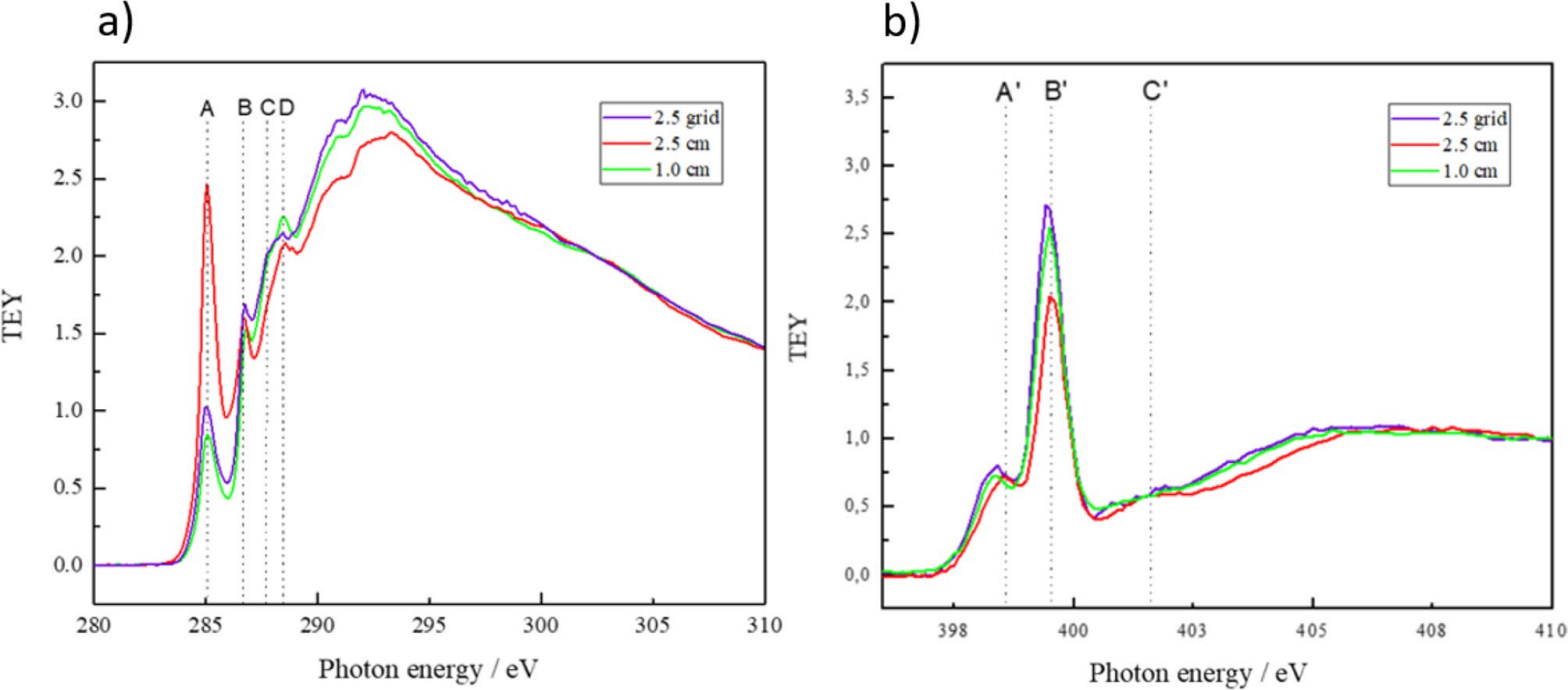
In (**a**) NEXAFS C K-edge and in (**b**) N K-edge spectra of samples placed under the 2.5 cm hole which is either covered with a grid (purple) or uncovered (red). The green line shows the spectrum of the sample placed under the hole of 1.0 cm diameter for comparison.

In principle, the filtering effects of the holes concern both, neutral radicals and ions. In order to study the contributions of ions to the deposition process a stainless steel grid with a transparency of 47% is placed on top of the grounded structured electrode. This grid acts as an electrode restricting the plasma to regions above the hole while allowing the flux of radicals to reach the substrate^64,65^: i.e., the grid preferentially blocks the contribution of the ions while it allows about 50% (equal to the transparency of the grid) of the radicals to pass it. (In the following experiment, the effect of the grid is only investigated for the hole with the largest diameter, 2.5 cm). The most obvious effect of the grid concerns the growth rate of the deposited films. The value of the growth rate exhibits a reduction by a factor higher than 10 when a grid is placed on the top of the electrode. NEXAFS C K-edge and N K-edge spectra in Fig. 5a,b show that also the film composition is affected by the presence of the grid.

In Fig. 5a the sample placed under the 2.5 cm hole with grid (purple line) shows a much smaller resonance intensity at 285 eV than the sample under the same 2.5 cm hole without grid (red line). This indicates a notable decrease of the aromatic characteristic of the film which comes along with an increase of the intensity of the σ* resonances around 294 eV underlining the stronger aliphatic feature of the film. It is worth to mention that the sample placed under the 2.5 cm hole with grid shows a similar behavior as the sample placed under the 1 cm hole without grid (green line in Fig. 5).

A similar trend can be observed for the N K-edge in Fig. 5b. The spectra of the sample placed under the 1 cm hole and the sample placed under the grid exhibit again a quite comparable behavior.

The results presented in Fig. 5 and the fact that the growth rate is substantially reduced due to the presence of the grid suggest that the ionic component plays an important role in the deposition process. There are two possible mechanisms that could explain the experimental findings: either the ions directly contribute to the growth of the film and are responsible for most of the film growth (see e.g. the discussion in^39,66,67^) or they activate the surface by creating dangling bonds which can then act as reaction sites either for the aniline molecule itself or for radicals created in the plasma. The latter, better known from plasma etching, is referred to as ion-neutral synergistic effect^42^.

In principle, the ion flux to the substrate -which consists mainly of positive aniline ions with a mass of 93 amu (see^28^) could be sufficiently high in our discharge to support the first mechanism. (Although the argon concentration is much larger than the aniline concentration in our experiment mass spectrometer measurements of positive ions^28^ show that the most abundant positive ion in our case is the aniline ion with a mass of 93 amu. (This can be explained by the fact that aniline has a much smaller ionization potential (7.7 eV) than argon (15.7 eV)). Measurements performed by means of microwave interferometry give a value of 5 × 10^15^ m^−3^ for the electron density. Taking this value, assuming an electron temperature even as low as 1 eV and applying the rather simple Bohm formula^39^:

$$\Gamma_{i} = 0.61 \cdot n_{i} \sqrt {\frac{{kT_{e} }}{{M_{i} }}}$$

gives a time averaged ion flux of about Γ_i_ = 1.5 × 10^18^ ions/(m^2^ s). Assuming a mass density even as large as 2 g/cm^3^ for the deposited thin films and a sticking coefficient of 1 results in a -purely ion supported- growth rate of about 8 nm/min which is about 2 times higher than the measured growth rate. (A discussion about the sticking coefficient of positive ions can be found e.g. in^39,40^). Even as this is twice the value of the observed growth rate it is important to stress, that this simple estimation does not rule out, that the ion induced surface activation (the second mechanism discussed above) does not play a role in the growth of our films.

## Conclusion

The aim of this work is to control the flux of different species that emerge from a reactive plasma and to control thus the properties of plasma deposited thin films. This is achieved by using a particular “channel electrode” design that is easy to implement in any given reactor for either scientific or industrial purpose. As a case study the deposition from an aniline/argon plasma is investigated.

The material characterization by means of FTIR and NEXAFS spectra shows that this electrode design effectively allows to tune the chemistry of the deposited material from highly aromatic to aliphatic amine. A similar result is observed by covering the electrode with a stainless steel grid, which is used to reduce preferentially the positive ion flux arriving on the substrate. This demonstrates the important role of positive ions for the deposition process in our particular setup and chemistry, affecting not only the deposition rate but also the composition of the deposited films.

It has to be mentioned, however, that the composition and magnitude of the neutral and ion fluxes for a given chemistry strongly depend on the discharge parameters. In particular, neutral radicals with a smaller sticking coefficient might be prone to losses due to pumping (a loss channel that depends on the pumping speed and on the actual volume of the process chamber and the plasma) and to losses due to electron impact fragmentation (a loss channel depending on electron temperature, electron density and residence time). Consequently, the role of neutral radicals and vice versa the role of the positive ions in the deposition process may strongly depend on the parameters and may therefore vary in different experiments.
